# Effect of microbial activity on penetrometer resistance and elastic modulus of soil at different temperatures

**DOI:** 10.1111/ejss.12440

**Published:** 2017-06-22

**Authors:** W. Gao, V. Muñoz‐Romero, T. Ren, R. W. Ashton, M. Morin, I. M. Clark, D. S. Powlson, W. R. Whalley

**Affiliations:** ^1^ Department of Soil and Water Sciences China Agricultural University No. 2 Yuanmingyuan West Road Beijing 100193 China; ^2^ Eco‐efficient Cropping Systems group, Departamento de Agronomía University of Cordoba Edificio C4 ‘Celestino Mutis’ Ctra. Madrid‐Cadiz km 396 14071 Cordoba Spain; ^3^ Sustainable Agriculture Sciences Department, Rothamsted Research West Common Harpenden AL5 2JQ UK

## Abstract

We explore the effect of microbial activity stimulated by root exudates on the penetrometer resistance of soil and its elastic modulus. This is important because it is a measure of the mechanical strength of soil and it correlates closely with the rate of elongation of roots. A sandy soil was incubated with a synthetic root exudate at different temperatures, for different lengths of time and with selective suppression of either fungi or bacteria. The shape of the temperature response of penetrometer resistance in soil incubated with synthetic exudate was typical of a poikilothermic temperature response. Both penetrometer resistance and small strain shear modulus had maximum values between 25 and 30°C. At temperatures of 20°C and less, there was little effect of incubation with synthetic root exudate on the small strain shear modulus, although penetrometer resistance did increase with temperature over this range (4–20°C). This suggests that in this temperature range the increase in penetrometer resistance was related to a greater resistance to plastic deformation. At higher temperatures (> 25°C) penetrometer resistance decreased. Analysis of the DNA sequence data showed that at 25°C the number of Streptomyces (Gram‐positive bacteria) increased, but selective suppression of either fungi or bacteria suggested that fungi have the greater role with respect to penetrometer resistance.

**Highlights:**

- Effect of microbial activity stimulated by synthetic root exudates on the mechanical properties.
- We compared penetrometer measurements and estimates of elastic modulus with microbial community.
- Penetrometer resistance of soil showed a poikilothermic temperature response.
- Penetrometer resistance might be affected more by fungi than bacteria.

## Introduction

Microbial activity stimulated by root exudates affects soil physical properties (Czarnes *et al*., 2000; Whalley *et al*., 2004; Barre & Hallett, 2009; Hinsinger *et al*., 2009). Biological exudates and their synthetic analogues have been shown to increase aggregate stability and tensile strength, and also alter soil hydraulic properties (Feeney *et al*., 2006; Ivanov & Chu, 2008; Hallett *et al*., 2013; Peng *et al*., 2011). Synthetic analogues of root exudates, such as polygalacturonic acid (PGA), have been shown to increase the elastic modulus of soil. Zhang *et al*. (2008) observed a doubling in the bond energy between 0 and 12 g PGA kg^−1^ soil. In a similar study, Chenu & Guerif (1991) found that briquettes of soil treated with scleroglucan, a fungal polysaccharide, greatly increased interparticle bonds. The microbial community can change considerably following the input of root exudates (Benizri *et al*., 2007; Broeckling *et al*., 2008). Much of the research on the link between microbial activity and soil strength is related to the elastic properties of soil, which is summarized in the review of Hallett *et al*. (2013). Penetrometer resistance, however, depends on a combination of both the plastic and elastic characteristics of soil (Farrell & Greacen, 1966; Ruiz *et al*., 2015). Penetrometer resistance is important because root elongation is very sensitive to soil strength, and penetrometer resistances in the region of 2.5 MPa, or more, have been shown to restrict root elongation (Bengough & Mullins, 1991). Microbial activity has a well‐defined temperature response curve; it increases initially with temperature to a maximum and then decreases with further increases in temperature. On the assumption that the bonding of soil particles by microbial exudates would increase penetrometer resistance, we were interested to determine whether penetrometer resistance also followed the temperature response curve associated with microbial activity.

Here we report the effect of incubation of soil with a synthetic root exudate described by Paterson *et al*. (2007). This was used because of the difficulty of extracting or harvesting natural root exudates. Soil amended with synthetic root exudate was incubated at a range of temperatures between 4 and 40°C together with a control. Following incubation, we measured the velocity of shear waves through the soil (Gao *et al*., 2013), which is related to the elastic properties of the soil fabric (Santamarina *et al*., 2001). We also measured penetrometer resistance with a needle penetrometer as described by Gao *et al*. (2013). At 4, 25 and 40°C extracted DNA was used to characterize the quantity and type of bacterial activity. We used selective biocides to determine the relative importance of fungal or bacterial activity with respect to penetrometer resistance. Our data provide new insights into the effects of microbial activity on soil strength.

## Materials and methods

### Experiment 1

A sample of loamy sand soil was taken from the 0–20‐cm soil layer at Butt Close field on Rothamsted Research's Woburn Experimental Farm in October, 2014. Sand, silt and clay contents are 88, 5 and 7 g 100 g^−1^, respectively. Soil organic matter content is 1 g 100 g^−1^. The soil was sieved through a 2‐mm sieve at the field water content at the time of collection. Large stones and plant residues were removed. The sieved soil was air‐dried to a water content of about 10 g 100 g^−1^, incubated at 25°C for 10 days and then stored in the dark at 4°C before use.

The solution of artificial root exudates was prepared following Paterson *et al*. (2007). It contained five sugars (glucose, sucrose, fructose, ribose and arabinose), five amino acids (glycine, valine, glutamine, serine and alanine) and five organic acids (malic acid, citric acid, malonic acid, oxalic acid and fumaric acid). Sufficient material to give 5.56 g carbon (C) of each compound was dissolved in 1000 ml of water. Three millilitres of this solution was mixed with 100 g dry soil to give a carbon addition rate of 250 mg per 100 g dry soil. The final soil water content was 15.5 g 100 g^−1^.

The soil, augmented with synthetic root exudate, was packed into a stainless ring (50‐mm high and 54 mm i.d.) with a 200 kPa axial pressure. This gave a soil bulk density of approximately 1.61 g cm^−3^. Control cores were packed at the same water content, but without any synthetic root exudate. All cores were incubated in the dark for 5 days at temperatures of 4, 10, 15, 20, 25, 30, 35 and 40°C. There were three replicates of each treatment combination. At the end of incubation period the soil cores were used to measure the penetrometer resistance and shear wave velocity. Water content, dry bulk density and microbial populations were also measured at the end of the incubation.

### Measurements of penetrometer resistance and shear wave velocity

The method for measuring penetrometer resistance is described in our previous papers (Gao *et al*., 2012a,2012b, 2013). The working system consisted of a 2‐mm diameter and 60° cone angle penetrometer with a 1.5‐mm shaft, a universal test frame (Davenport–Nene Test Frame DN10, Wigston, UK) and an electronic balance to measure the penetrometer force. The penetrometer was inserted into the soil core at a constant speed of 20 mm minute^−1^ from the surface to a depth of 50 mm. The force needed to push the penetrometer into the soil was recorded with an electronic balance which supported the core. Three penetrometer measurements were made on each soil core.

Shear wave velocity, *V*_s_, was measured on each soil core with a piezo‐ceramic device at the top and bottom of the soil samples (Gao *et al*., 2013), then the penetrometer measurements were made. The time taken for an S wave (shear wave) to travel the length of the core was determined by comparing the input signal to the soil core with the detected wave (Whalley *et al*., 2011, 2012). The sensors, related electronics and software are available commercially (GDS Instruments, 32 Murrell Green Business Park, London Road, Hook, UK, RG27 9GR).

After completing the measurements, the soil cores were weighed, then oven‐dried for 24 hours at 105°C to determine water content and bulk density.

### Measurements of microbial population

We quantified the microbial population with the method described by Hirsch *et al*. (2016). Microbial DNA was extracted from 250 mg soil for each replicate (three replicates per treatment) with the PowerSoil^®^ DNA isolation kit (Mo Bio Laboratories, Inc., Carlsbad, CA, USA) by following the manufacturer's instructions, except for the beading step which used 5.5 m s^−1^ for 30 s (FastPrep^®^ 24 Instrument, MP Biomedicals, Santa Ana, CA, USA). The quality and quantity of the DNA were checked with a NanoDrop Spectrophotometer (NanoDrop products, Wilmington, DE, USA) (200–320 nm) and the Qubit quantification platform with Quant^®^ dsDNA BR Assay kit (molecular probes^®^ Life Technologies, Thermo Fisher Scientific, Waltham, MA, USA), respectively. Amplification of bacterial and archaeal 16S rRNA genes for high‐throughput Illumina MiSeq (Illumina, San Diego, CA, USA) sequencing and microbial community composition (UPARSE + QIIME analysis) were done following Pylro *et al*. (2014).

### Experiment 2: selective suppression of bacteria and fungi

In this experiment soil was prepared for incubation as described above, but either a bacteriocide (2‐Bromo‐2‐nitropropane‐1,3‐diol (Bronopol)) or a fungicide (Captan 80WDG) was added to exudate‐treated soil as additional treatments. They were added at 0.135 and 0.53 g 100 g^−1^ soil (Lin & Brookes, 1999). The effects of these were compared to a control sample prepared exactly as described in Experiment 1, and with the same water content. To investigate the effects of time, samples with added exudate and control samples were incubated for 1, 3, 5, 10, 15 20 and 30 days. Penetrometer resistance and shear wave velocity were measured as described above.

### Statistical design

The treatments were randomized and repeated three times. Each of these replicate cores was treated independently. The measurements were taken at the end of the incubation periods. We analysed the data with an analysis of variance (anova) as a randomized block design experiment and plotted the mean data together with the least significant difference (LSD) for *P* = 0.05 (LSD *P* = 0.05). Experiment 1 had two levels of exudate (+ or −) at each of eight incubation temperatures, giving 16 treatment combinations with a treatment structure of ‘exudate (+ or −) × temperature’ with a block structure of ‘soilcore/sample’, where ‘/’ indicates that samples were taken independently from the soil cores. Experiment 2 had two levels of exudate (+ or −) and three treatments to suppress microbial activity (Bronopol, Captan 80WDG or water) and eight incubation temperatures, giving (2 × 3 × 8) 48 treatment combinations, with a treatment structure of ‘exudate (+ or −) × microbial suppressant (Bronopol, Captan 80WDG or water) × temperature’. The block structure in Experiment 2 was ‘soilcore/sample’. When the effects of time were investigated there were two levels of exudate (+ or −) and seven time periods, giving 14 treatment combinations with the treatment structure of ‘exudate (+ or −) × days of incubation’ and a block structure of ‘soilcore/sample’. In all three analyses we found the interaction was significant at *P* < 0.001, and plotted the appropriate LSD (*P* = 0.05) to allow these comparisons to be made.

Microbial composition was assessed in a duplicate experiment. In this experiment we plotted the data from the different replicate cores.

## Results

### Experiment 1

**Figure 1:**
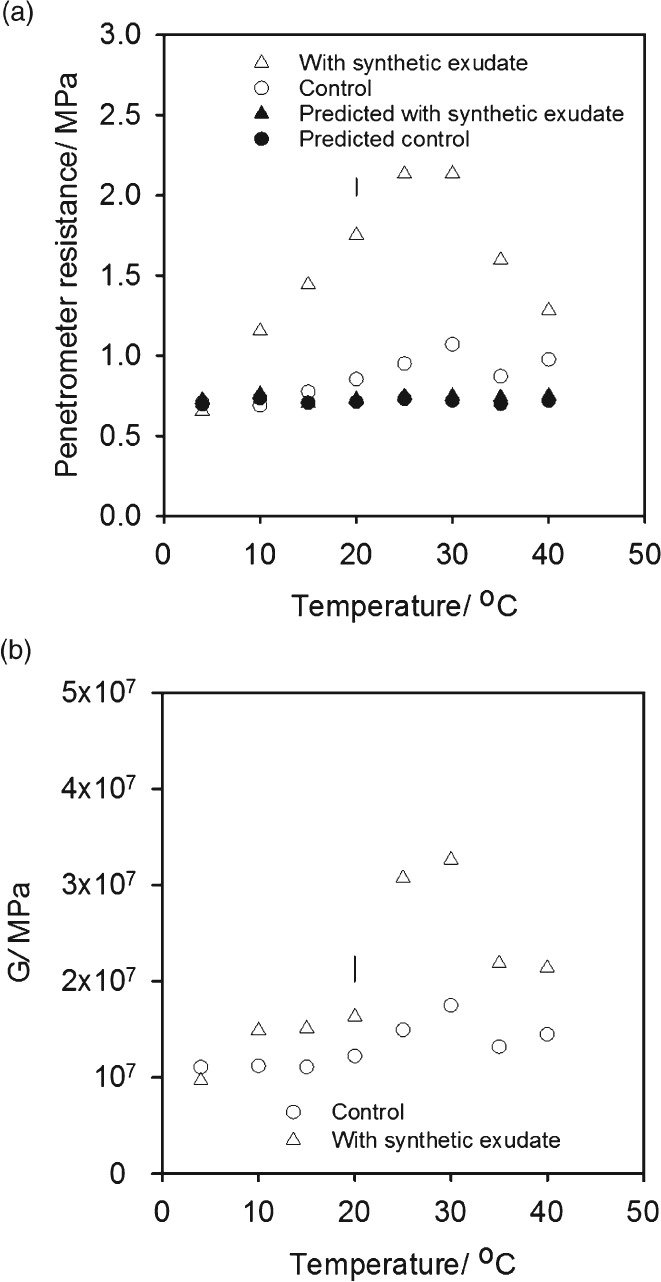
(a) Penetrometer resistance of soil amended with a synthetic mixture of sugars, amino acids and organic acids designed to replicate root exudate and a control soil plotted as a function of temperature and (b) the small strain shear modulus for soil amended with synthetic exudate and a control soil plotted as a function of temperature. The LSD for P = 0.05 is plotted. Part (a) shows the predicted penetrometer resistance calculated with the model of Gao et al. (2012b); appropriate bulk density and water content data were used.

The temperature response of penetrometer resistance in the amended soil was consistent with an expected poikilothermic temperature response of the soil microbial community (Figure 1a), where biological activity depends on the external temperature of the organism. Penetrometer resistance showed a gradual increase with temperature up to 25°C, followed by a decrease between 30 and 40°C. When soil with the addition of artificial root exudate was incubated at between 4 and 20°C there was a small increase only in the small strain shear modulus (Figure 1b). At incubation temperatures of 25 and 30°C the shear modulus increased considerably with addition of the artificial root exudate, but the difference was much less at the two highest temperatures (Figure 1b). In summary, the patterns of dependence of penetrometer resistance and small strain modulus on temperature were different in detail, but they did show some similarities. At 4°C, where microbial activity would be expected to be small, neither penetrometer resistance nor small strain shear modulus were affected by the treatment with exudate. Similarly at 40°C, considered to be above the optimum temperature for temperate oceanic environmental microbial activity, the effect of added root exudate was again relatively small for both measurements. Both, however, were affected considerably by the addition of synthetic exudate in the range of temperatures likely to favour microbial activity.

**Figure 2:**
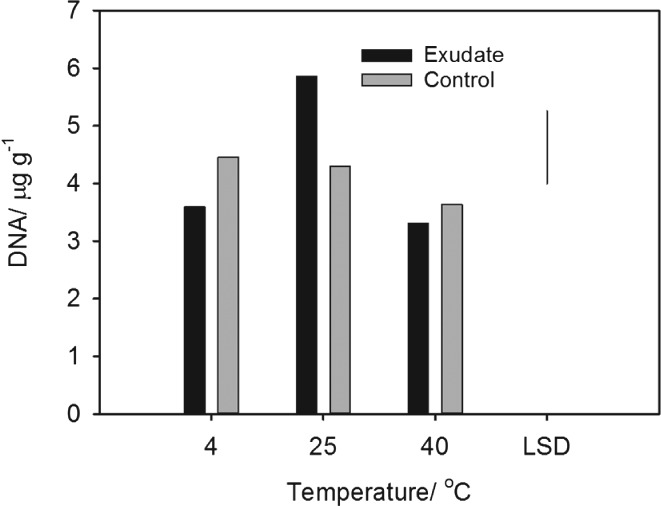
The concentration of DNA in soil incubated with synthetic exudate compared with the control. The LSD for P = 0.05 is plotted.

**Figure 3:**
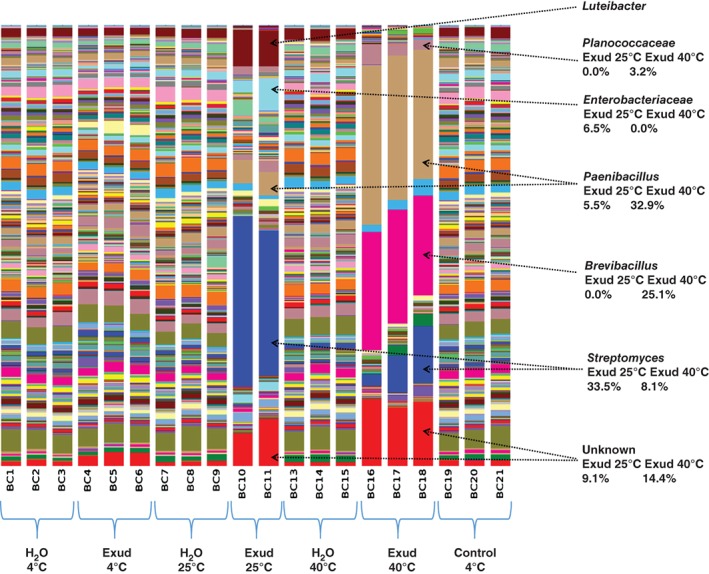
Bacterial composition of soil samples inferred from the analysis of DNA. Percentage abundance of 16S rRNA amplicons for selected groups are displayed. Different bars for the same temperature treatment represent replicates. Exud, Exudate.

**Figure 4:**
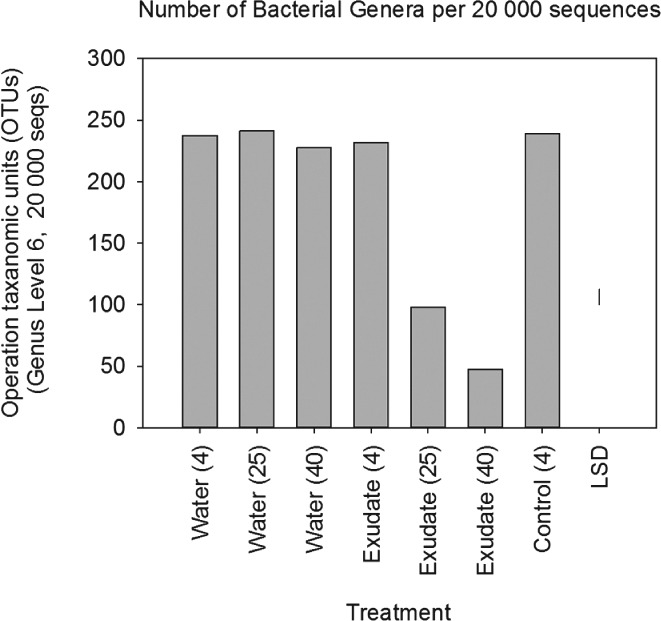
Number of bacterial and archaeal operational taxonomic units (OTUs) (genera) per 20 000 amplicon sequences per replicate. The LSD for P = 0.05 is plotted. The temperature of incubation (in °C) is shown in brackets. Incubation of the control is at 4°C without the addition of either water or exudate.

The total amount of DNA extracted from the soil (Figure 2) corresponds to the expected effects of temperature on microbial growth, with the largest increase from exudate addition at 25°C. At 25°C the soil treated with synthetic exudate had a much greater proportion of *Streptomyces* than the untreated soil, which is shown by 16s bacterial RNA (Figure 3). At 40°C this trend was also apparent, but less marked; at 4°C there was little difference between treated and untreated soil in relation to bacterial RNA. At 25°C there was slightly more *Luteibacter* in the treated soil. At 40°C the numbers of *Paenibacllilus* and *Brevibacllilus* increased in exudate‐treated soil, although these changes at the highest temperature did not appear to be reflected in penetrometer resistance or soil rigidity (Figure 1). Figures 3 and 4 also show the effect that root exudates have at higher temperatures (25 and 40°C) on the selection for dominant microbial genera, and on the decrease in overall diversity compared to the control and water treatments.

**Figure 5:**
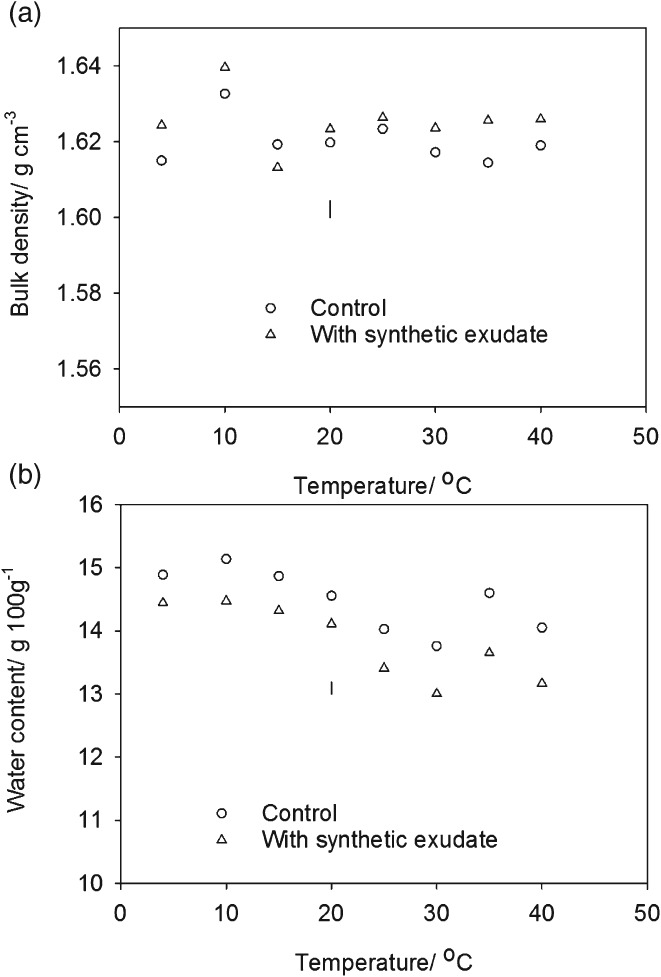
(a) Bulk density and (b) water content of the soil samples following application of the treatments. The LSD for P = 0.05 is plotted.

**Figure 6:**
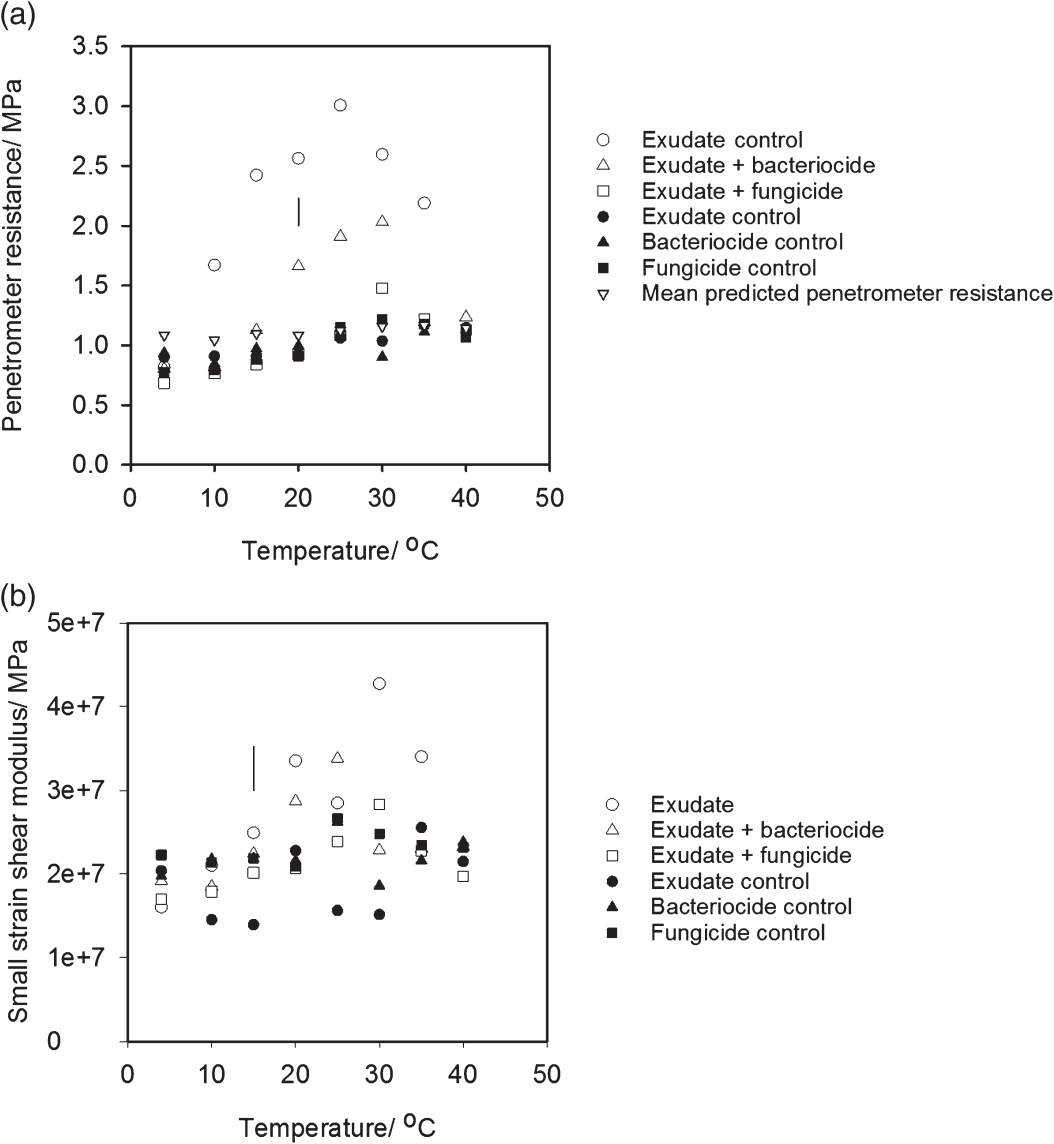
(a) The effect of selective suppression of microbial communities on penetrometer resistance and (b) the small strain shear modulus as a function of temperature. These soil samples were incubated for 10 days. Part (a) shows the predicted penetrometer resistance calculated with the appropriate bulk density and water content data, and the model of Gao et al. (2012b). The LSD for P = 0.05 is plotted.

Figure 5 shows that neither water content nor bulk density differed greatly with temperature and exudate treatments. This is important because they provide alterative explanations for the observed increases in penetrometer resistance and small strain shear modulus. Although cores treated with a synthetic exudate were slightly drier at all temperatures (approximately 0.5 g 100 g^−1^), this difference was too small to account for the differences in shear wave velocity (i.e. small strain shear modulus) or penetrometer resistance (Whalley *et al*., 2012; Gao *et al*., 2012b, 2013). At 4°C there was no effect of exudate treatment despite a small reduction in water content (14.9–14.4 g 100 g^−1^). This provides experimental evidence that these small differences in water content did not account for the increases in differences in shear wave velocity or penetrometer resistance. Over a wider range of soil water contents (15.1–12.9 g 100 g^−1^), the effect on penetrometer resistance can be estimated with the empirical model of Gao *et al*. (2012b), which was parameterized for the soil used in this study. In Figure 1 (and Figure 6) we have plotted penetrometer resistance predictions at different temperatures using the water content and soil density data in Figure 5. The central point is that the differences in water content shown in Figure 5 did not affect matric potential sufficiently to change penetrometer resistance. In both Experiments 1 and 2, the predictions of penetrometer resistance at low and high temperatures agreed with the experimental data. Larger penetrometer resistances were predicted in Experiment 2 than in Experiment 1, which was caused by a slightly larger soil bulk density (1.75 g cm^−3^ compared to 1.60 g cm^−3^) in Experiment 2. Penetrometer resistance showed no marked dependence on temperature for both experiments when the water content data in Figure 5 and water release characteristics of a similarly packed soil were used.

### Experiment 2

**Figure 7:**
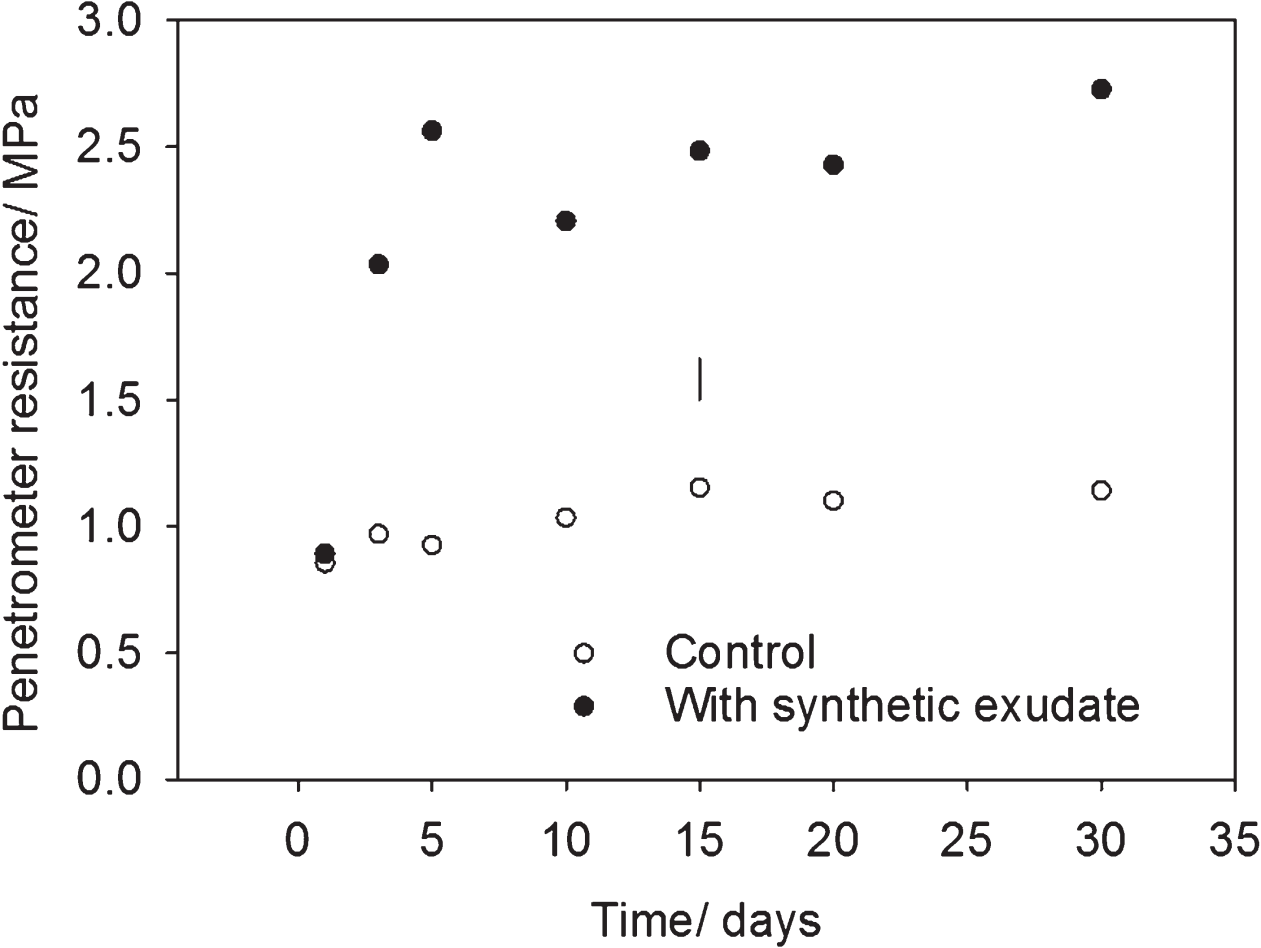
The effect of incubation time on penetrometer resistance of soil incubated at 20°C. The LSD for P = 0.05 is plotted.

The results obtained in the first experiment (Figure 1) were replicated in the second experiment (Figure 6). Furthermore, these data indicated that the suppression of fungal activity by fungicide had the most marked effect on penetrometer resistance. When the temperature was greater than 15°C, penetrometer resistance in the bacteriocide treatment was greater than that under the fungicide treatment. The second experiment also showed that at low temperatures (less than 20°C) there was little increase in the elastic strength of the soil, which was inferred from the estimates of small strain shear modulus, despite an increase in the penetrometer resistance. The increase in penetrometer resistance persisted for at least 30 days (Figure 7).

## Discussion

In this study, we added low molecular weight compounds to soil designed to simulate the effects of root exudates on microbial activity. Although these compounds can affect pH, which can alter the mechanical properties of clay soil (Tarchitzky & Chen, 2002), these additions in a sand dominated soil are unlikely to affect the strength of the soil fabric; however, they stimulate the soil's microbial population to produce exudates of uncertain composition. Inter‐particle bonding mediated by extracellular polysaccharides and fungal hyphae can reinforce the soil fabric. Tisdall *et al*. (2012) suggested that the movement of particles held together by the fungus *Mucor* sp. led mainly to ductile failure of a disc of soil under tensile stress, whereas metabolites or dispersed clay on the surfaces of hyphae limited deformation. One of the most striking observations from our data was that at temperatures between 10 and 20°C there was a progressive increase in penetrometer resistance in exudate‐treated soil that was not accompanied by an increase in the small strain shear modulus of soil (Figure 1). The strain applied to the soil in the measurement of the small strain shear modulus from shear wave velocity was extremely small, and an increase in shear wave velocity reflected an increase in the elastic strength of the bonds between soil particles. At between 25 and 30°C there was a significant increase (*P* < 0.001) in both small strain shear modulus and also penetrometer resistance. This suggests that at temperatures between 10 and 20°C, the larger penetrometer resistance is related to an increase in the ability of soil to resist plastic deformation. However, between 25 and 40°C an increase in the elastic strength of soil was at least partly responsible for the greater penetrometer resistance compared to that of the control treatment. This supports the proposal by Tisdall *et al*. (2012), that various biological process can give rise to a range of different elastic strengths depending on the fungal species, the nature of the bond or both. A comparison of the temperature responses in Figure 1 suggests that when substrates (root exudates) are added the temperature response of the penetrometer resistance follows closely the expected growth rates of microbial populations (Pietikäinen *et al*., 2005), with the optimal growth and maximum penetrometer resistance between 25 and 30°C. Above this range, rates of bacterial and fungal growth are expected to decline in the same way that penetrometer resistance does. Different mechanisms appear to be responsible for the increased penetrometer resistance at 20°C and below; no increase in elastic strength was detected compared to temperatures greater than 20°C when both the elastic strength increased together with penetrometer resistance.

Analysis of the extracted DNA showed that one of the effects of incubation with root exudate at 25and 40°C was an increase in the number of *Streptomyces,* which are filamentous bacteria. The increase in *Streptomyces* was particularly large at 25°C. It is probable that they contribute to both the increase in small strain shear modulus and penetrometer resistance at these temperatures. Despite the coincidence of the raised values of penetrometer resistance and small strain shear modulus, and more *Streptomyces*, the selective suppression of bacteria and fungi (Figure 6) showed that soil fungi made the greater contribution to increased penetrometer resistance, although suppression might not have been complete. The data in Figure 6 support the result from Experiment 1 that at low temperatures (less than 20°C) the increases in penetrometer resistance (between 4 and 20°C) were not related to any change in the elastic properties of the soil. For the most common soil temperatures, especially at depth (i.e. less than 25°C), soil fungi might be the most important microbiological factors that determine penetrometer resistance. Rousk *et al*. (2008) demonstrated that Bronopol inhibited bacterial growth to < 1% of the rate of growth compared to controls when Bronopol was added at 320 µg g^−1^ soil. Bronopol and Captan are likely to have only partial inhibition of soil microbial populations, therefore further analysis is required to confirm the role of bacteria and fungi in penetrometer resistance. The maximum small shear strain modulus from the contribution of fungi appears to be at a lower temperature than for bacteria (approximately 25°C for fungi and 30°C for bacteria; Figure 6). At present the DNA fungal data base is too limited to provide any meaningful dissection of the contributions from the different fungal groups (P. Hirsch, personal communication).

The increase in penetrometer resistance resulting from the addition of artificial root exudate appeared to occur within the first day when incubated at 20°C, and it persisted for at least 30 days (Figure 7). These data show some consistency with the temporal effect of glucose on aggregate stability summarized by Tisdall & Oades (1982). They proposed that aggregate stability increases for up to 3 or 4 weeks following the addition of glucose and the production of polysaccharides. Cosentino *et al*. (2006) showed an increase in rates of respiration following the addition of straw to soil after 2 days, and the persistence of increased water repellency for up to 63 days. Future research should develop our results further, which showed an increase in penetrometer resistance with time, to characterize both the decline and increase in penetrometer resistance with time.

## Conclusions

We have demonstrated that in soil amended with synthetic root exudate and then incubated, penetrometer resistance was a function of the temperature of incubation. The shape of the temperature response was typical of a poikilothermic temperature response. At temperatures of 20°C and lower, there was little effect on the small strain shear modulus of incubation with synthetic root exudate, although penetrometer resistance did increase with temperature over this range (4–20°C). Analysis of the DNA sequence data showed that at 25°C there were increased numbers of the bacteria *Streptomyces*, whereas selective suppression of either fungi or bacteria suggested that fungi might have the greater role with respect to penetrometer resistance.
